# The determinants of COVID-19 vaccine uptake among migrants from 109 countries residing in China: A cross-sectional study

**DOI:** 10.3389/fpubh.2022.1023900

**Published:** 2023-01-16

**Authors:** Hao Chen, Weitian Lei, Zhengyi Wei, Fan Wang

**Affiliations:** ^1^Department of Preventive Medicine and Health Education, School of Public Health, Fudan University, Shanghai, China; ^2^School of Politics and International Relations, East China Normal University, Shanghai, China; ^3^Fudan Development Institute, Fudan University, Shanghai, China

**Keywords:** COVID-19, vaccine uptake, foreign migrants, vaccine hesitancy, health equality

## Abstract

**Background:**

The present study aimed to investigate the prevalence of COVID-19 vaccine uptake among foreign migrants in China and to explore the determinants of their vaccine uptake behavior.

**Methods:**

From June to October 2021, we used convenience and snowball sampling to recruit a sample of 764 participants from five cities in which the overwhelming majority of foreign migrants in China live. The chi-square (χ^2^) tests were used to examine vaccination distribution according to demographic characteristics. Multivariate logistic regression models visualized by forest plot were used to investigate the associations between significant determinants and vaccine uptake.

**Results:**

Overall, the prevalence of vaccination rate was 72.9% [95% confidence interval (CI): 69.9–76.0%]. Migrants whose social participation was very active [adjusted odds ratio (AOR): 2.95, 95% CI: 1.36–6.50, *P* = 0.007] or had perceived COVID-19 progression prevention by the vaccine (AOR: 1.74, 95% CI: 1.01–3.02, *P* = 0.012) had higher odds of vaccination compared to those whose social participation was inactive or who did not have this perception. Migrants who perceived the vaccine uptake process as complex (AOR: 0.47, 95% CI: 0.27–0.80, *P* = 0.016) or were unsure of their physical suitability for the vaccine (AOR: 0.40, 95% CI: 0.24–0.68, *P* < 0.001) had lower odds of vaccination compared to those who did not have these perceptions. Furthermore, migrants from emerging and developing Asian countries (AOR: 2.32, 95% CI: 1.07–5.21, *P* = 0.04) and the Middle East and Central Asia (AOR: 2.19, 95% CI: 1.07–4.50, *P* = 0.03) had higher odds of vaccination than those from major advanced economies (G7) countries, while migrants from other advanced economic countries (OR: 0.27, 95% CI: 0.11–0.63, *P* = 0.003) had lower odds of vaccination than those from G7 countries.

**Conclusion:**

It may be beneficial to promote vaccine uptake among migrants by ensuring effective community engagement, simplifying the appointment and uptake process, and advocating the benefits and target populations of the COVID-19 vaccine.

## Introduction

According to the data from the International Organization for Migration (IOM), there were ~281 million international migrants worldwide in 2020, equivalent to 3.6% of the global population (1). Migrant health is public health, and available mortality data show that migrants from low/middle-income countries to Europe and the USA have higher excess COVID-19 mortality, compared to native citizens (2, 3). Thus, ensuring the COVID-19 vaccination of these populations is essential for all destination countries (4). Even before the COVID-19 pandemic, migrants were considered in danger of underimmunization (5, 6). Migrants face well-documented barriers to accessing healthcare, such as some European countries restricting access to vaccination initiatives for certain groups of migrants (7). Furthermore, language barriers and social exclusion also lead to their mistrust of vaccine uptake (5, 8). The WHO recommends that the distribution of COVID-19 vaccines should give priority to marginalized refugees and migrants and call on all populations to have affordable and non-discriminatory access to vaccines (9).

In recent decades, China has increasingly become an important destination country for migrants worldwide: in 2017, ~1 million international migrants were registered in China (10). As an essential part of global health, which China has promised to promote since the Sustainable Development Goals (SDGs) were signed at the UN General Assembly in September 2015 (11), the health problems of foreign migrants have provoked public concerns in China. During the early outbreak of COVID-19 in 2020, China's Ministry of Foreign Affairs responded that China always attaches high importance to the wellbeing of foreign migrants and has taken effective measures to address their concerns and needs (12). However, few countries, including China, have measured the vaccination situation, and how personal, social, and policy barriers or facilitators influence vaccine uptake among migrants (13, 14). Those studies suggested the determinants of COVID-19 vaccine uptake among foreign migrants not only include unawareness of the importance of COVID-19 vaccine uptake, doubting vaccine safety, and effectiveness but also include having difficulty in access to media coverage about vaccine messaging, proficiency in the English language and supportive policy or planning, especially those who are low-skilled labor migrants, refugees, undocumented migrants could have low access to vaccination lived in Europe countries (13, 14).

The 5As taxonomy is defined as a complex mix of demographic, structural, social, and behavioral factors that are considered to contain most determinants of vaccine uptake (8, 15). These determinants (the 5 “A's”) include (1) access, which refers to the ability to access vaccines, such as native language proficiency and confidence in policy-makers or government; (2) affordability, which refers to the ability of individuals to afford vaccination, both in terms of financial and non-financial costs, such as direct vaccine costs and time costs; (3) awareness, which refers to the degree to which individuals have knowledge of the need for vaccines and risks and entitlement to knowledge about vaccination and risks; (4) acceptance, which refers to the degree to which individuals accept, question, or refuse vaccination, such as perceived vaccine safety, side effects, and benefits; and (5) activation, the degree to which individuals are encouraged to be vaccinated, such as vaccination incentives and health education (8). Regarding access, inequalities in access to COVID-19 vaccination by undocumented migrants (16) and historical experiences of migrants influence COVID-19 vaccine uptake (17). Language barriers and lack of interpreting services were common barriers to measles and hepatitis B vaccine uptake (18, 19). Acceptance of vaccines was found in the perceived importance and effectiveness of vaccination and low perceived risk of vaccine-preventable diseases (20, 21). Kathleen found that the vaccination demand of undocumented migrants in Switzerland, the USA, Italy, and France was only 41.2%, whereas they found that the affordability dimension, including sources of information about COVID-19, and the awareness dimension, including positive views on COVID-19 vaccination, influenced the demand for vaccination (22). Regarding activation, personalized vaccination reminders had a larger positive effect on the uptake of childhood vaccines (23), and health promotion helped promote vaccination in communities that had experienced measles outbreaks (24). Although the 5As taxonomy is suitable for explaining direct and potential influencing factors of vaccine uptake among migrants, this taxonomy is rarely comprehensively applied to COVID-19 vaccination, especially for foreign migrants residing in China.

Therefore, how high are vaccination coverage and what are determinants of vaccine uptake from the 5As perspective in the migrant population in China are significant research questions that should be explored.

## Methods

### Study population

**Figure 1:**
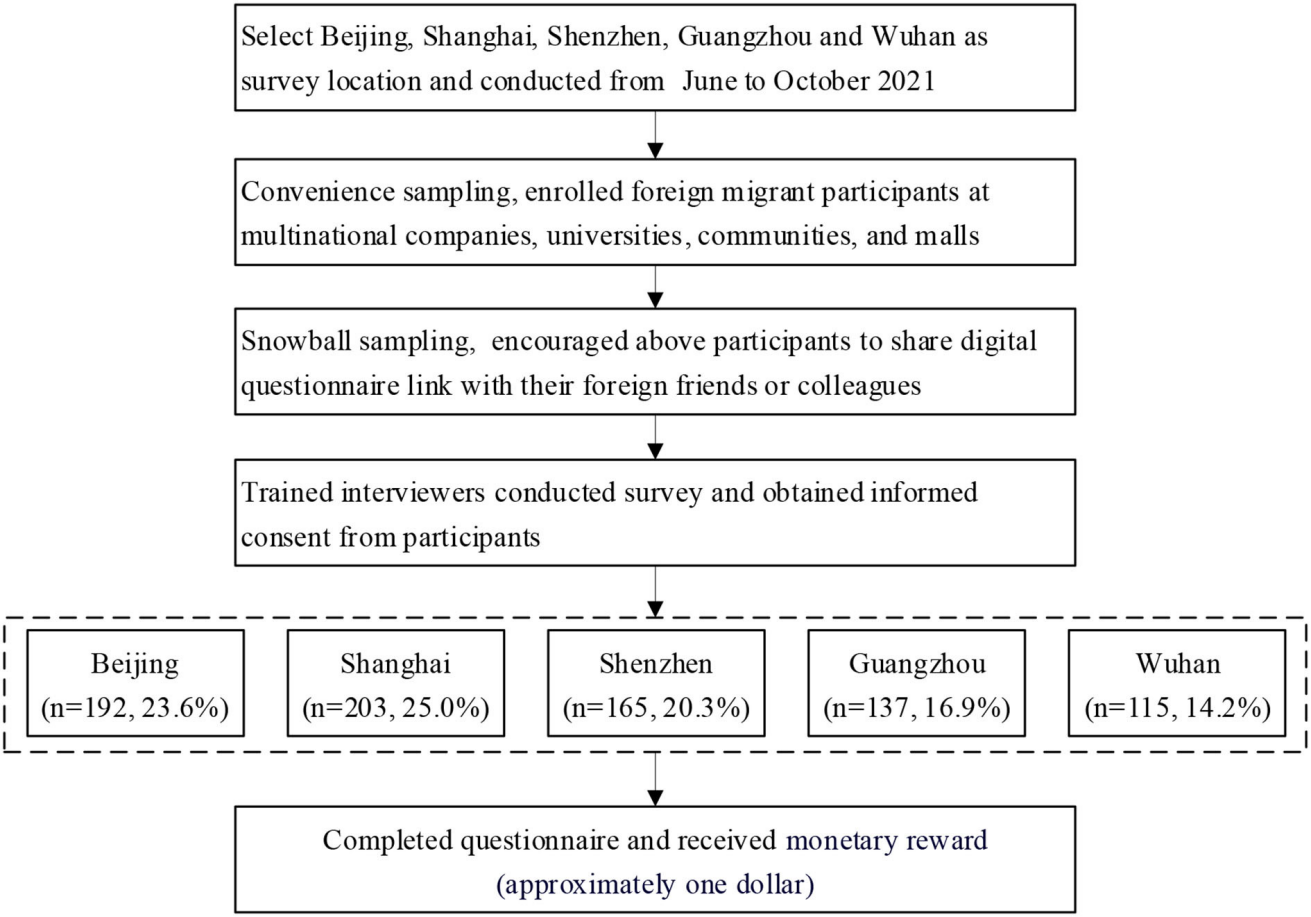
The flow chart of data collection and survey.

From June to October 2021, convenience and snowball sampling were used to recruit a sample of 812 participants from Beijing, Shanghai, Shenzhen, Guangzhou, and Wuhan, which are the cities with the overwhelming majority of foreign migrants in China. According to China's 7th National Census Data (25), Guangdong is the province with the largest foreign population, and Shenzhen and Guangzhou are the largest cities. At the same time, the number of foreign people in Shanghai and Beijing ranked third and fifth in this national census. In addition, Wuhan was the worst-struck city when COVID-19 broke out in 2020. Hence, the aforementioned five cities were selected. The convenience sampling method was conducted by trained interviewers who invited foreign migrant participants to complete a digital questionnaire at multinational companies, universities, communities, and malls with migrants. The price of one dose of the COVID-19 vaccine is ~US$15 for foreign migrants in Beijing (26), Shanghai (27), Guangdong (28), Zhejiang (29), and Wuhan (30). The snowball sampling method was conducted by the participants as mentioned previously, who were encouraged to share digital questionnaire links with their foreign friends or colleagues. Each participant received a small monetary reward (~1 dollar) after authentically completing the digital questionnaire. The process of data collection and survey are shown in Figure 1. The minimum sample size was calculated to be 475 by using the following formula: $deff\times \frac{Z_{1-\alpha /2}^{2}p\left(1-p\right)}{d^{2}}$, where reported prevalence of COVID-19 vaccine uptake rate (p) was 19.1% (31). The type I error (α) was 0.05 thus z_1−α/2_ = 1.96, the precision (d) was 0.05, and the design effect (deff) was 2 (32). The inclusion criteria for participants' enrolment were as follows: (1) aged over 16 years, (2) foreign migrants who were living or traveling in China, and (3) able to understand the English or Chinese version of the questionnaire by themselves. All participants provided their online written informed consent before the survey was conducted. The present study was approved by the Institutional Review Board of the East China Normal University Committee on Human Research Protection (HR 161-2021).

### COVID-19 vaccine uptake and its determinants

COVID-19 vaccine uptake was assessed using a single question: “Have you ever received the COVID-19 vaccine? (yes/no)” The determinants of the COVID-19 vaccine were adopted from a previous study which contained five essential dimensions as follows (8):

(1) Access consisted of six dimensions: (a) Chinese language proficiency, which was assessed by single questions: “What is your Chinese language level?” Participants were required to answer “basic,” “intermediate,” or “advanced.” (b) Multilingual service in vaccination, which was assessed by the single question “The lack of multilingual services when making appointments and getting Chinese coronavirus vaccines caused me inconvenience (yes/no).” (c) Trust in the Chinese government, which was assessed by the question “How confident are you in the Chinese government's fight against the pandemic? (scored from 0 to 100).” (d) Social participation was assessed by seven questions, including precautions, offline fundraising to help pandemic-stricken areas, offline volunteer activities for epidemic prevention and control, obtaining COVID-19-related information proactively, proactively posting COVID-related information, participating in an online fundraiser to help affected areas, and engaging in online volunteering. Each item was scored on a five-point Likert scale, ranging from 1 (“never or very seldom”) to 5 (“very often”). We divided the mean score of social participation into three levels, which were “inactive (<2),” “moderately active (2–3),” and “very active (4–5).” € The Chinese vaccine policy benefits the public, which was assessed by the single question “Do you agree with the Chinese vaccine policy benefits to the public (yes/no).” (f) Vaccination in the local district was assessed by the single question “Can you receive COVID-19 vaccination in the local district (yes/no).” The Cronbach's alpha of the access dimension was 0.818.
(2) Affordability consisted of seven dimensions: (a) Vaccine price, which was assessed by the question “Do you think COVID-19 vaccine prices are acceptable (expensive/inexpensive)?” (b) Adequate vaccination sites, which were assessed by the question “COVID-19 vaccination sites are conveniently located in shopping malls, office buildings, and subway exits (yes/no).” (c) Long queues for vaccination, which was assessed by the question “I experienced or know there was a long queue for COVID-19 vaccination (yes/no).” (d) Complexity in vaccine appointments and uptake, which was assessed by the question “The process of making an appointment and receiving the COVID-19 vaccine is complicated (yes/no).” (e) Trust in Chinese media, which was assessed by the question “Do you trust Chinese media coverage about COVID-19? (yes/no).” (f) Usefulness of Chinese media, which was assessed by the question “Do you feel that Chinese media coverage on COVID-19 is useful to you? (yes/no).” (g) Promptness of Chinese social media, which was assessed by the question “Did you feel that Chinese social media coverage on COVID-19 is timely? (yes/no).” The Cronbach's alpha of the affordability dimension was 0.701.
(3) Awareness consisted of six items: (a) Unsure of physical suitability for vaccine, which was assessed by the question “I am unsure of my physical suitability for the COVID-19 vaccine (yes/no).” (b) Perceived susceptibility to COVID-19 assessed by the question “I am at risk of COVID-19 (low/high).” (c) Perceived severity of COVID-19, which was assessed by the question “The consequences of getting COVID-19 are severe (low/high).” (d) Confirmed COVID-19 cases in the community, which was assessed by the question “Are there confirmed COVID-19 cases in your community? (yes/no).” (e) Confirmed COVID-19 cases among friends, which was assessed by the question “Are there confirmed COVID-19 cases among your friends? (yes/no).” (f) Confirmed COVID-19 infection, which was assessed by the question “Have you ever had COVID-19? (yes/no).” The Cronbach's alpha of the awareness dimension was 0.746.
(4) Acceptance consisted of six items: (a) Doubt regarding the safety of the vaccine, which was assessed by the question “I am doubtful of the safety of the COVID-19 vaccine.” (b) Perceived instant side effects of the vaccine, which were assessed by the question “I am worried about the instant side effects of the COVID-19 vaccine.” (c) Perceived long-term side effects of the vaccine, which were assessed by the question “The consequences of getting COVID-19 are severe.” (d) Perceived effectiveness of the vaccine, which was assessed by the question “I am worried about the long-term side effects of the COVID-19 vaccine.” (e) Preventing COVID-19 infection by vaccination, which was assessed by the question “The COVID-19 vaccine is effective in preventing COVID-19 infection (yes/no).” (f) Preventing the progression of COVID-19 to severe disease by vaccination, which was assessed by the question “The COVID-19 vaccine is effective in preventing the progression of COVID-19.” Each item was scored on a five-point Likert scale, ranging from 1 (“strongly disagree”) to 5 (“strongly agree”). Participants who answered 4 (“agree”) and 5 (“strongly agree”) were classified into the positive group, namely, the “high” or “yes” group, while the others were classified into the negative group, namely, the “low” or “no” group. The Cronbach's alpha of the acceptance dimension was 0.914.
(5) Activation consisted of two items: (a) Acceptability of gifts for vaccination, which was assessed by the question “Do you believe that sending a gift before COVID-19 vaccination is beneficial for promoting vaccination? (yes/no).” (b) Acceptability of advertising for vaccination, which was assessed by the question “Do you believe that advertising the COVID-19 vaccine is beneficial for promoting vaccination? (yes/no).” Cronbach's alpha of the activation dimension was 0.741. According to the result of Harman's one-factor test, the aforementioned five dimensions suggested there is no variance method bias because the first factor loading of the five dimensions is <40% (33).

### Adjustment variables

Adjusted variables in the present study included age, sex, nationality (34), educational attainment, religious beliefs, annual income (RMB), occupation, living status, years of living in China, and whether the respondent stayed in China during the outbreak (from January 2020 to March 2020). Nationality comprised countries of seven economic levels, which included major advanced economies (G7 countries, e.g., USA and Japan), other advanced economies (e.g., Australia and Iceland), European area countries (e.g., the Netherlands and Estonia), emerging and developing Asian countries (e.g., India and Myanmar), emerging and developing European countries (e.g., Albania and Belarus), Latin America and Caribbean countries (e.g., Argentina and Chile), Middle Eastern and Central Asian countries (e.g., Afghanistan and Iran), and sub-Saharan African countries (e.g., Angola and Chad), sorted by gross domestic product (GDP) level.

### Statistical analysis

First, we used descriptive analysis to show the characteristics of participants, and the chi-square (χ^2^) tests were used to examine the distribution of vaccination according to demographic characteristics. Furthermore, we also used the χ^2^-tests to explore the potential determinants of vaccine uptake. Finally, multivariate logistic regression models visualized by forest plots were used to examine the associations between significant determinants and vaccine uptake after adjusting for significant characteristics from χ^2^-tests. The estimates of determinants for COVID-19 vaccine uptake were summarized using odds ratios (OR) and their 95% confidence intervals (CI). Two-tailed analyses calculated *P-*values, with *P* < 0.05 considered statistically significant. All statistical analyses were performed using R software (version 4.1.1) (35).

### Patient and public involvement statement

No patients were involved in this study.

## Results

### Participant characteristics

The questionnaires of participants who met the following exclusion criteria were discarded: (1) Chinese nationality (*n* = 24), (2) no specific nationality information (*n* = 6), and (3) returned invalid questionnaires (*n* = 18). Finally, 764 participants were included in this study with a valid questionnaire rate of 94.1%.

As shown in Table 1, the present study included 764 participants from 109 countries aged between 17 and 71 years (mean age 29.28, standard deviation (SD) 8.27); 67.8% were male. Most of the participants were from middle-income countries (56.5%), had religious beliefs (78.8%), were students (62.7%), and stayed in China during the COVID-19 outbreak (84.4%). Slightly less than half of the participants reported an annual income (44.1%) lower than 50,000 RMB, having a bachelor's degree (43.7%), living alone in China (49.9%), and living in China for 1–3 years (44.8%). Detailed information on the distribution of age, sex, and vaccination rate according to each country is presented in Supplementary Table 1.

**Table 1:** Vaccination rates and their distribution by demographic characteristics.

**Variable**	***N* (%)**	**Vaccination**		**χ^2^, *P*-value**
		**No**	**Yes**	
**Age**				9.038, 0.029
17–24	228 (29.8)	73 (32.0)	155 (68.0)	
25–34	392 (51.3)	100 (25.5)	292 (74.5)	
35–44	98 (12.4)	34 (23.6)	110 (76.4)	
45–71	49 (6.5)	6 (12.2)	43 (87.8)	
**Gender**				2.376, 0.123
Male	518 (67.8)	131 (25.3)	387 (74.7)	
Female	246 (32.2)	76 (30.9)	170 (69.1)	
**Nationality**				30.233, <0.001
Low-income	136 (17.8)	48 (35.3)	88 (64.7)	
Lower-middle income	280 (36.6)	45 (16.1)	235 (83.9)	
Upper-middle income	152 (19.9)	43 (28.3)	109 (71.7)	
High-income	196 (25.7)	71 (36.2)	125 (63.8)	
**Country groups**				57.958, <0.001
Euro area	20 (2.7)	5 (25.0)	15 (75.0)	
Major advanced economies (G7)	120 (15.7)	31 (25.8)	89 (74.2)	
Other advanced economies	46 (6.0)	31 (67.4)	15 (32.6)	
Emerging and developing Asia	98 (12.8)	16 (16.3)	82 (83.7)	
Emerging and developing Europe	36 (4.7)	13 (36.1)	23 (63.9)	
Latin America and the Caribbean	75 (9.8)	22 (29.3)	53 (70.7)	
Middle east and central Asia	166 (21.7)	26 (16.3)	82 (83.7)	
Sub-Saharan Africa	203 (26.6)	63 (31.0)	140 (69.0)	
**Education attainment**				21.398, <0.001
≤Middle school	61 (8.0)	24 (39.3)	37 (60.7)	
Bachelor's degree	334 (43.7)	103 (30.8)	231 (69.2)	
Master's degree	260 (34.0)	68 (26.2)	192 (73.8)	
Doctor's degree	109 (14.3)	12 (11.0)	97 (89.0)	
**Religious belief**				2.295, 0.130
No	162 (21.2)	52 (32.1)	110 (67.9)	
Yes	602 (78.8)	155 (25.7)	447 (74.3)	
**Annual income**				5.285, 0.152
<5	337 (44.1)	92 (27.3)	245 (72.7)	
5–15	154 (20.2)	51 (33.1)	103 (66.9)	
16–35	176 (23.0)	44 (25.0)	132 (75.0)	
>35	97 (12.7)	20 (20.6)	77 (79.4)	
**Occupation**				0.148, 0.929
Student	479 (62.7)	132 (27.6)	347 (72.4)	
Profit job	204 (26.7)	54 (26.5)	150 (73.5)	
Non-profit job	81 (10.6)	21 (25.9)	60 (74.1)	
**Living status**				1.200, 0.273
Cohabitation	383 (50.1)	111 (29.0)	272 (71.0)	
Living alone	381 (49.9)	96 (25.2)	285 (74.8)	
**Years for living in China**				3.673, 0.159
1–3	342 (44.8)	82 (24.0)	260 (76.0)	
4	208 (27.2)	58 (27.9)	150 (72.1)	
5	214 (28.0)	67 (31.3)	147 (68.7)	
**Stay in China during outbreak**				0.914, 0.339
No	119 (15.6)	37 (31.1)	82 (68.9)	
Yes	645 (84.4)	170 (26.4)	475 (73.6)	

### Univariate analysis for vaccination distribution

Overall, the prevalence of vaccination was 72.9% (95% CI: 69.9–76.0%). As shown in Table 1, the results of χ^2^-tests suggested a significant difference in the prevalence of vaccination for nationality and educational attainment (*P* < 0.001). As shown in Table 2, among the access determinants of vaccine uptake, there was a statistically significant difference between Chinese language proficiency (χ^2^ = 10.102, *P* < 0.001) and social participation (χ^2^ = 7.254, *P* = 0.027). The prevalence of vaccination among participants who perceived that the Chinese vaccine policy benefited the public (76.8%) was higher than that among participants who did not perceive that the Chinese vaccine policy benefited the public (63.5%; χ^2^ = 10.102, *P* < 0.001). Among affordability determinants, there was a higher prevalence of vaccination among participants who perceived adequate vaccination sites (76.9 vs. 66.4%, *P* = 0.002), no complexity in vaccine appointments or uptake (74.4% vs. 63.1%, *P* = 0.016), trust in Chinese media (75.5 vs. 68.7%, *P* = 0.048), and promptness of Chinese media (75.6 vs. 67.9%, *P* = 0.030). Among awareness determinants, participants who were unsure of their physical suitability for the vaccine showed a lower vaccination prevalence (76.7 vs. 57.6%, *P* < 0.001). Among acceptance determinants, those participants who perceived instant side effects of the vaccine (63.2 vs. 75.2%, *P* = 0.005) and perceived long-term side effects of the vaccine (66.3 vs. 74.7%, *P* = 0.040) had a lower prevalence of vaccination than those participants who without these perceptions. Those participants who believed in the effectiveness of the vaccine (78.2 vs. 59.8%, *P* < 0.001), perceived the prevention of COVID-19 infection by the vaccine (77.5 vs. 65.3%, *P* < 0.001), and perceived the prevention progression of COVID-19 to severe disease by the vaccine (78.3 vs. 58.5%, *P* < 0.001) showed a higher prevalence of vaccination than participants without these perceptions. However, the association between activation determinants and vaccination was not significant.

**Table 2:** Vaccination rates and their distribution by 5As model components.

**Variable**	***N* (%)**	**Vaccination**		**χ^2^, *P*-value**
		**No**	**Yes**	
**Access**				
Chinese langue proficiency				10.102, 0.006
Basic	318 (41.6)	70 (22.0)	248 (78.0)	
Intermediate	320 (41.9)	91 (28.4)	229 (71.6)	
Advanced	126 (16.5)	46 (36.5)	80 (63.5)	
Multilingual service in vaccination				0.338, 0.561
No	158 (22.6)	47 (29.7)	111 (70.3)	
Yes	541 (77.4)	146 (27.0)	395 (73.0)	
Trust in Chinese government				5.467, 0.065
Low trust (<60)	115 (15.1)	39 (33.9)	76 (66.1)	
Trust (60–90)	282 (37.1)	82 (29.1)	200 (70.9)	
Very trust (>90)	364 (48.8)	86 (23.6)	278 (76.4)	
Social participation				7.254, 0.027
Inactive	105 (13.7)	35 (33.3)	70 (66.7)	
Moderately active	533 (69.8)	149 (28.0)	384 (72.0)	
Very active	126 (16.5)	23 (18.3)	103 (81.7)	
Chines vaccine policy benefits to public				13.313, <0.001
No	222 (29.1)	81 (36.5)	141 (63.5)	
Yes	542 (70.9)	126 (23.2)	416 (76.8)	
Vaccination at local district				0.393, 0.531
No	115 (16.5)	35 (30.4)	80 (69.6)	
Yes	584 (83.5)	158 (27.1)	426 (72.9)	
**Affordability**				
Vaccine price				0.528, 0.467
Acceptable	589 (84.3)	159 (27.0)	430 (73.0)	
Expensive	110 (15.7)	34 (30.9)	76 (69.1)	
Adequate vaccination sites				9.485, 0.002
No	292 (38.2)	98 (33.6)	194 (66.4)	
Yes	472 (61.8)	109 (23.1)	363 (76.9)	
Long queues for vaccination				0.196, 0.658
No	535 (76.5)	145 (27.1)	390 (72.9)	
Yes	164 (23.5)	48 (29.3)	116 (70.7)	
Complex in vaccine appointment and uptake				5.811, 0.016
No	577 (82.5)	148 (25.6)	429 (74.4)	
Yes	122 (17.5)	46 (36.9)	77 (63.1)	
Trust in Chinese media				3.926, 0.048
No	294 (38.5)	92 (31.3)	202 (68.7)	
Yes	470 (61.5)	115 (24.5)	355 (75.5)	
Usefulness of Chinese media				0.087, 0.768
No	309 (40.4)	86 (27.8)	223 (72.2)	
Yes	455 (59.6)	121 (26.6)	334 (73.4)	
Promptness of Chinese social media				4.718, 0.030
No	265 (34.7)	122 (24.4)	377 (75.6)	
Yes	499 (65.3)	85 (32.1)	180 (67.9)	
**Awareness**				
Unsure of physical suitability for vaccine				21.319, <0.001
No	613 (80.2)	143 (23.3)	470 (76.7)	
Yes	151 (19.8)	64 (42.4)	87 (57.6)	
Perceived susceptibility to COVID-19				1.454, 0.228
Low	635 (83.1)	166 (26.1)	469 (73.9)	
High	129 (16.9)	41 (31.8)	88 (68.2)	
Perceived severity to COVID-19				0.765, 0.382
Low	249 (32.6)	73 (29.3)	176 (70.7)	
High	515 (67.4)	134 (26.0)	381 (74.0)	
Confirmed COVID-19 case in community				2.160, 0.117
No	608 (79.6)	173 (28.5)	435 (71.5)	
Yes	156 (20.4)	34 (21.8)	122 (78.2)	
Confirmed COVID-19 case among friends				2.701, 0.100
No	540 (70.7)	156 (28.9)	384 (71.1)	
Yes	224 (29.3)	51 (22.8)	173 (77.2)	
Confirmed COVID-19 infected				<0.001, 1
No	746 (98.4)	202 (27.1)	544 (72.9)	
Yes	12 (1.6)	3 (25.0)	9 (75.0)	
**Acceptance**				
Doubt on safety of vaccine				1.571, 0.210
No	653 (85.5)	171 (26.2)	482 (73.8)	
Yes	111 (14.5)	36 (32.4)	75 (67.6)	
Perceived instant side effect of vaccine				7.897, 0.005
No	620 (81.2)	154 (24.8)	466 (75.2)	
Yes	144 (18.8)	53 (36.8)	91 (63.2)	
Perceived long-term side effect of vaccine				4.218, 0.040
No	601 (78.7)	152 (25.3)	449 (74.7)	
Yes	163 (21.3)	55 (33.7)	108 (66.3)	
Perceived effectiveness of vaccine				25.703, <0.001
No	219 (28.7)	88 (40.2)	131 (59.8)	
Yes	545 (71.3)	119 (21.8)	426 (78.2)	
Prevent COVID-19 infected by vaccine				12.831, <0.001
No	285 (37.3)	99 (34.7)	186 (65.3)	
Yes	479 (62.7)	108 (22.5)	371 (77.5)	
Prevent progression of COVID-19 to severe by vaccine				29.024, <0.001
No	207 (27.1)	86 (41.5)	121 (58.5)	
Yes	557 (72.9)	121 (21.7)	436 (78.3)	
**Activation**				
Acceptable of gifts for vaccination				2.509, 0.113
No	383 (50.1)	114 (29.8)	269 (70.2)	
Yes	381 (49.9)	93 (24.4)	288 (75.6)	
Acceptable of advertising for vaccination				2.778, 0.096
No	272 (35.6)	84 (30.9)	188 (69.1)	
Yes	492 (64.4)	123 (25.0)	369 (75.0)	

### Multivariate analysis of the association between vaccination and its determinants

**Figure 2:**
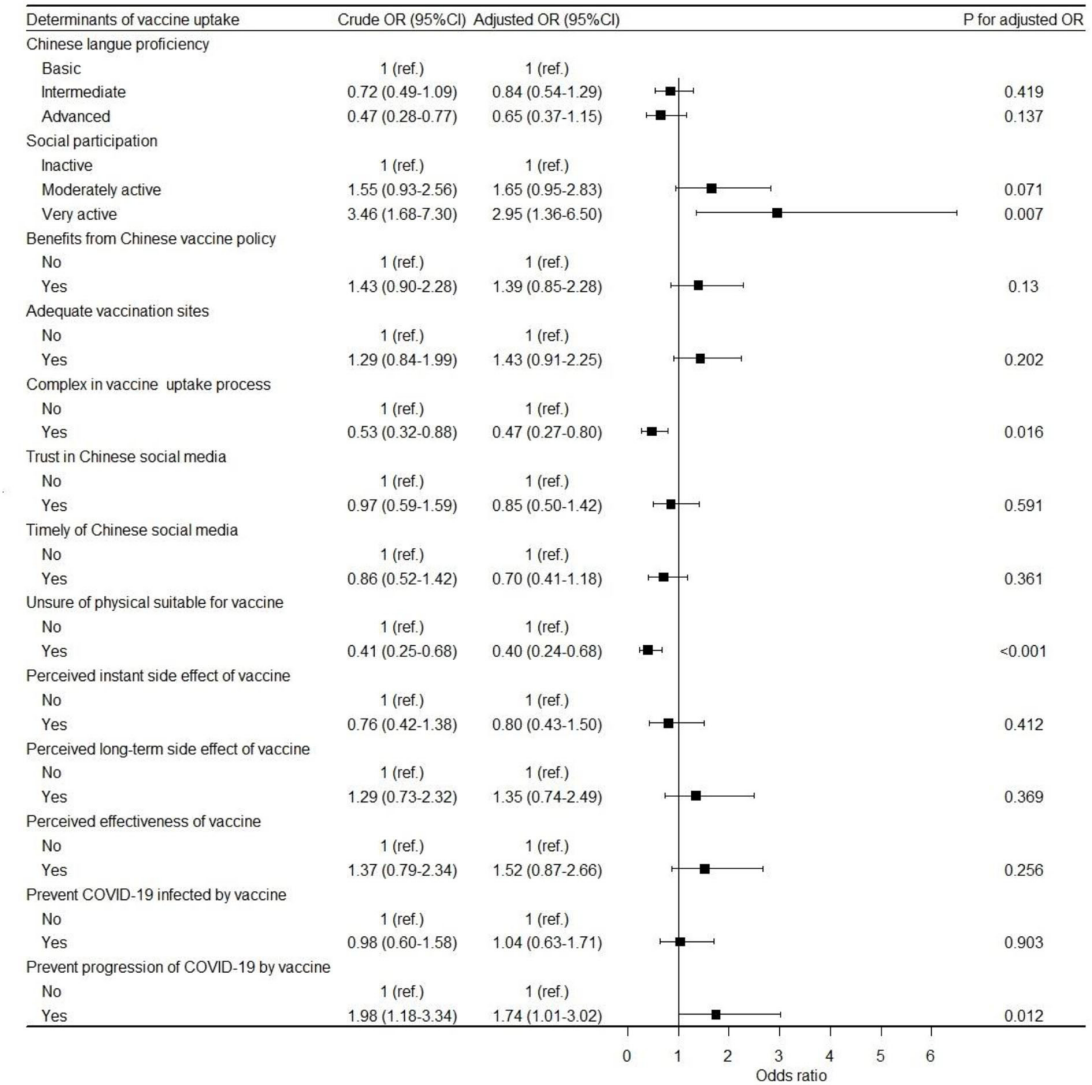
Forest plot of determinants of vaccine uptake among migrants in China.

The results of the crude and adjusted multivariate logistic regression models for associations of determinants with the odds of vaccination are shown in Figure 2. After adjusting for nationality and educational attainment, migrants whose social participation was very active [adjusted odds ratio (AOR): 2.95, 95% CI: 1.36–6.50, *P* = 0.007] or who perceived that the vaccine prevented the progression of COVID-19 (AOR: 1.74, 95% CI: 1.01–3.02, *P* = 0.012) had higher odds of vaccination compared to those whose social participation was inactive or those who did not have this perception. Migrants who perceived that the vaccine uptake process was complex (AOR: 0.47, 95% CI: 0.27–0.80, *P* = 0.016) or were unsure of their physical suitability for the vaccine (AOR: 0.40, 95% CI: 0.24–0.68, *P* < 0.001) had lower odds of vaccination compared to those who did not have these perceptions. Furthermore, migrants from emerging and developing Asian countries (AOR: 2.32, 95% CI: 1.07–5.21, *P* = 0.04) and Middle Eastern and Central Asian countries (AOR: 2.19, 95% CI: 1.07–4.50, *P* = 0.03) had higher odds of vaccination than those from G7 countries, while migrants from other advanced economic countries (AOR: 0.27, 95% CI: 0.11–0.63, *P* = 0.003) had lower odds of vaccination than those from G7 countries.

### Cluster analysis of the association between vaccination and its determinants according to age and sex

The determinants of COVID-19 vaccination according to sex are shown in Table 3. Among male participants, migrants whose social participation was moderately active (AOR: 2.12, 95% CI: 1.07–4.18, *P* = 0.029) and very active (AOR: 4.35, 95% CI: 1.68–11.70, *P* = 0.002) had higher odds of vaccination than those whose social participation was inactive. The migrants who believed that the vaccine appointment process was complex (AOR: 0.37, 95% CI: 0.19–0.72, *P* = 0.003) and felt unsure of their physical suitability for vaccination (AOR: 0.35, 95% CI: 0.19–0.64, *P* < 0.001) had lower odds than those who did not believe. Migrants aged between 45 and 71 years had higher odds (AOR: 4.58, 95% CI: 1.18–24.25, *P* = 0.043) of vaccination than those aged 17–24 years. Among female participants, migrants aged between 35 and 44 years had higher odds (AOR: 0.23, 95% CI: 0.09–6.86, *P* = 0.032) of vaccination than those aged 17–24 years. Compared to those with middle school education, participants with a bachelor's degree (AOR: 7.33, 95% CI: 2.48–23.06, *P* < 0.001), master's degree (AOR: 10.50, 95% CI: 2.85–42.34, *P* < 0.001), or doctoral degree (AOR: 10.85, 95% CI: 1.41–115.95, *P* = 0.030) had higher odds of vaccination. Migrants from emerging and developing Asian countries (AOR: 6.75, 95% CI: 1.48–37.56, *P* = 0.019) had higher odds of vaccination than those from G7 countries, while migrants from other advanced economic countries (AOR: 0.17, 95% CI: 0.04–0.63, *P* = 0.011) had lower odds of vaccination than those from G7 countries.

**Table 3:** Determinants of vaccination rates for the components of the 5As models according to sex.

	**Male**			**Female**		
	***N***	**OR (95% CI)**	***P*** **-value**	***N***	**OR (95% CI)**	***P*** **-value**
**Age**						
17–24	136 (26.3)	1 (ref.)		92 (37.4)	1 (ref.)	
25–34	284 (54.8)	0.98 (0.52–1.82)	0.937	108 (43.9)	0.71 (0.28–1.75)	0.456
35–44	62 (12.0)	0.82 (0.35–1.95)	0.648	33 (36.4)	0.23 (0.06–0.86)	0.032
45–71	36 (6.9)	4.58 (1.18–24.25)	0.043	13 (5.3)	0.69 (0.09–6.66)	0.727
**Education attainment**						
≤Middle school	28 (5.4)	1 (ref.)		33 (13.4)	1 (ref.)	
Bachelor's degree	209 (40.3)	0.65 (0.20–1.93)	0.461	125 (50.8)	7.33 (2.48–23.06)	<0.001
Master's degree	72 (29.3)	0.61 (0.18–1.91)	0.413	72 (29.3)	10.50 (2.85–42.34)	<0.001
Doctor's degree	16 (6.5)	1.37 (0.34–5.18)	0.649	16 (6.5)	10.85 (1.41–115.95)	0.030
**Country groups**						
Major advanced economies (G7)	77 (14.9)	1 (ref.)		43 (17.5)	1 (ref.)	
Other advanced economies	16 (3.1)	1.56 (0.37–8.42)	0.364	30 (12.2)	0.17 (0.04–0.63)	0.011
Euro area	15 (2.9)	0.55 (0.15–2.00)	0.566	5 (2.0)	2.06 (0.21–48.87)	0.573
Emerging and developing Asia	67 (12.9)	1.84 (0.69–5.02)	0.228	31 (12.6)	6.75 (1.48–37.56)	0.019
Emerging and developing Europe	22 (4.2)	0.59 (0.18–2.00)	0.382	14 (5.7)	0.79 (0.17–3.81)	0.768
Latin America and the Caribbean	24 (4.6)	0.75 (0.23–2.51)	0.632	51 (20.7)	2.80 (0.87–9.47)	0.088
Middle east and central Asia	137 (26.4)	1.85 (0.76–4.46)	0.171	29 (11.8)	3.58 (0.78–19.16)	0.115
Sub–Saharan Africa	160 (30.9)	0.93 (0.42–2.01)	0.855	43 (17.5)	2.52 (0.70–9.93)	0.169
**Chinese langue proficiency**						
Basic	228 (44.0)	1 (ref.)		90 (36.5)	1 (ref.)	
Intermediate	216 (41.7)	0.87 (0.51–1.48)	0.598	104 (42.3)	1.03 (0.42–0.63)	0.947
Advanced	74 (14.3)	0.51 (0.25–1.05)	0.064	52 (21.1)	0.69 (0.23–2.54)	0.512
**Social participation**						
Inactive	71 (13.7)	1 (ref.)		34 (13.8)	1 (ref.)	
Moderately active	358 (69.1)	2.12 (1.07–4.18)	0.029	175 (71.1)	1.03 (0.42–3.48)	0.758
Very active	89 (17.2)	4.35 (1.68–11.70)	0.002	37 (15.0)	0.69 (0.23–8.43)	0.496
**Chines vaccine policy benefits to public**						
No	150 (29.0)	1 (ref.)		72 (29.3)	1 (ref.)	
Yes	368 (71.0)	1.60 (0.92–2.79)	0.080	174 (70.7)	0.99 (0.34–2.85)	0.991
**Complex in vaccine appointment and uptake**						
No	397 (83.1)	1 (ref.)		180 (81.4)	1 (ref.)	
Yes	81 (16.7)	0.37 (0.19–0.72)	0.003	41 (18.6)	0.51 (0.17–1.51)	0.220
**Trust in Chinese media**						
No	186 (35.9)	1 (ref.)		108 (43.9)	1 (ref.)	
Yes	332 (64.1)	0.88 (0.45–1.70)	0.711	138 (56.1)	0.46 (0.15–1.27)	0.142
**Promptness of Chinese social media**						
No	163 (31.5)	1 (ref.)		102 (41.5)	1 (ref.)	
Yes	355 (68.5)	0.66 (0.34–1.27)	0.221	144 (58.5)	0.92 (0.29–2.90)	0.886
**Unsure of physical suitability for vaccine**						
No	412 (79.5)	1 (ref.)		201 (81.7)	1 (ref.)	
Yes	106 (20.5)	0.35 (0.19–0.64)	<0.001	45 (18.2)	0.92 (0.28–3.13)	0.896
**Perceived instant side effect of vaccine**						
No	425 (82.0)	1 (ref.)		195 (79.3)	1 (ref.)	
Yes	93 (18.0)	1.01 (0.46–2.25)	0.994	51 (20.7)	0.36 (0.10–1.27)	0.115
**Perceived long–term side effect of vaccine**						
No	413 (79.7)	1 (ref.)		188 (76.4)	1 (ref.)	
Yes	105 (20.3)	1.59 (0.75–3.45)	0.234	58 (23.6)	0.73 (0.23–2.40)	0.604
**Perceived effectiveness of vaccine**						
No	144 (27.8)	1 (ref.)		75 (30.5)	1 (ref.)	
Yes	374 (72.2)	1.61 (0.79–3.28)	0.191	171 (69.5)	1.29 (0.43–3.84)	0.651
**Prevent COVID**−**19 infected by vaccine**						
No	192 (37.1)	1 (ref.)		64 (26.0)	1 (ref.)	
Yes	326 (62.9)	0.90 (0.48–1.66)	0.740	182 (74.0)	1.27 (0.45–3.53)	0.650
**Prevent progression of COVID**−**19 to severe by vaccine**						
No	143 (27.6)	1 (ref.)		93 (37.8)	1 (ref.)	
Yes	375 (72.)	1.44 (0.71–2.88)	0.309	153 (62.2)	4.36 (1.41–14.77)	0.013

The determinants of COVID-19 vaccination according to age are shown in Table 4. Among 17–27-year-old migrants, migrants from Middle Eastern and Central Asian countries (AOR: 5.32, 95% CI: 1.25–23.91, *P*=0.026) and sub-Saharan Africa (AOR: 4.97, 95% CI: 1.25–23.91, *P* = 0.026) had higher odds of vaccination than those from G7 countries. Migrants who believed that the vaccine prevented the progression of COVID-19 to severe disease showed higher odds of vaccination than those who did not believe (AOR: 3.93, 95% CI: 1.35–12.30, *P* = 0.014). Among 25–34-year-old migrants, those with a bachelor's degree (AOR: 9.78, 95% CI: 2.07–52.63, *P* = 0.005), master's degree (AOR: 6.96, 95% CI: 1.49–37.01, *P* = 0.016), or doctoral degree (AOR: 17.35, 95% CI: 2.94–117.73, *P* = 0.002) had higher odds of vaccination than migrants who had a middle school education or below. Migrants from other advanced economic countries (AOR: 0.17, 95% CI: 0.04–0.63, *P* = 0.007) had lower odds of vaccination than those from G7 countries, while those from emerging and developing Asian countries (AOR: 4.97, 95% CI: 1.25–23.91, *P* = 0.026) showed higher odds of vaccination than those from G7 countries. Migrants who felt unsure of their physical suitability for vaccination (AOR: 0.26, 95% CI: 0.11–0.58, *P* = 0.001) had lower odds than those who were sure. Among the 17–24- and 35–71-year-old participants, migrants whose social participation was moderately active (AOR: 1.66, 95% CI: 1.44–5.81, *P* = 0.029; OR: 2.44, 95% CI: 1.57–10.54, *P* = 0.012) and very active (AOR: 3.04, 95% CI: 1.64–14.68, *P* = 0.002; OR: 6.82, 95% CI: 2.54–140.56, *P* = 0.002) had higher odds of vaccination than those whose social participation was inactive. Migrants who believed that the vaccine uptake process was complex (AOR: 0.17, 95% CI: 0.04–0.64, *P* = 0.004; AOR: 0.12, 95% CI: 0.02–0.65, *P* = 0.017) had lower odds of vaccination than those without this perception.

**Table 4:** Determinants of vaccination rates for the components of 5As models according to age.

	**17–24 years old**			**25–34 years old**			**35–71 years old** ^*^		
	***N***	**OR (95% CI)**	***P*** **-value**	***N***	**OR (95% CI)**	***P*** **-value**	***N***	**OR (95% CI)**	***P*** **-value**
**Sex**									
Male	136 (59.6)	1 (ref.)		284 (72.4)	1 (ref.)		98 (68.1)	1 (ref.)	
Female	92 (40.4)	1.45 (0.63–3.51)	0.391	108 (27.6)	1.23 (0.62–2.51)	0.456	46 (31.9)	0.33 (0.08–1.28)	0.055
**Education attainment**									
≤Middle school	40 (17.5)	1 (ref.)		12 (3.1)	1 (ref.)		9 (6.3)	1 (ref.)	
Bachelor's degree	168 (73.7)	1.40 (0.48–3.94)	0.022	129 (32.9)	9.78 (2.07–52.63)	0.005	37 (25.7)	3.22 (0.30–34.29)	0.322
Master's degree	19 (8.3)	1.25 (0.25–6.86)	0.257	180 (45.9)	6.96 (1.49–37.01)	0.016	61 (42.4)	4.15 (0.40–41.44)	0.221
Doctor's degree	1 (0.4)	-	-	71 (18.1)	17.35 (2.94–117.73)	0.002	37 (25.7)	2.95 (0.21–42.48)	0.415
**Country groups**									
Major advanced economies (G7)	21 (9.2)	1 (ref.)		54 (13.8)	1 (ref.)		45 (31.3)	1 (ref.)	
Other advanced economies	20 (8.7)	0.24 (0.04–1.20)	0.091	17 (4.3)	0.17 (0.04–0.63)	0.007	9 (6.3)	0.41 (0.04–3.65)	0.423
Euro area	3 (1.3)	-	-	8 (2.0)	2.06 (0.21–48.87)	0.573	9 (6.3)	3.27 (0.28–69.83)	0.391
Emerging and developing Asia	34 (14.9)	3.87 (0.90–17.39)	0.071	51 (13.0)	6.75 (1.48–37.56)	0.033	13 (9.0)	0.21 (0.02–3.50)	0.237
Emerging and developing Europe	8 (3.5)	0.52 (0.03–5.46)	0.606	17 (4.3)	0.79 (0.17–3.81)	0.484	11 (7.6)	0.57 (0.05–10.32)	0.677
Latin America and the Caribbean	26 (11.4)	0.75 (0.23–2.51)	0.302	35 (8.9)	2.80 (0.87–9.47)	0.831	14 (9.7)	1.81 (0.22–17.37)	0.589
Middle east and central Asia	34 (14.9)	5.32 (1.25–23.91)	0.026	113 (28.8)	3.58 (0.78–19.16)	0.173	19 (13.2)	0.31 (0.04–2.08)	0.236
Sub-Saharan Africa	82 (36.0)	4.97 (1.26–20.07)	0.022	97 (24.7)	2.52 (0.70–9.93)	0.141	24 (16.7)	5.38 (0.76–50.82)	0.112
**Chinese langue proficiency**									
Basic	47 (20.6)	1 (ref.)		189 (48.2)	1 (ref.)		82 (56.9)	1 (ref.)	
Intermediate	131 (57.5)	1.12 (0.42–2.88)	0.810	151 (38.5)	0.84 (0.45–1.57)	0.586	38 (26.4)	0.43 (0.12–1.58)	0.126
Advanced	50 (21.9)	0.51 (0.25–1.05)	0.396	52 (13.3)	0.58 (0.24–1.44)	0.228	24 (16.7)	0.65 (0.11–1.28)	0.153
**Social participation**									
Inactive	19 (8.3)	1 (ref.)		62 (15.8)	1 (ref.)		24 (16.7)	1 (ref.)	
Moderately active	165 (72.4)	1.66 (1.44–5.81)	0.029	264 (67.3)	1.55 (0.72–3.17)	0.255	104 (72.2)	2.44 (1.57–10.54)	0.012
Very active	44 (19.3)	3.04 (1.64–14.68)	0.002	66 (16.8)	2.55 (0.80–8.50)	0.118	16 (11.1)	6.82 (2.54–140.56)	0.002
**Chines vaccine policy benefits to public**									
No	78 (34.2)	1 (ref.)		112 (28.6)	1 (ref.)		32 (22.2)		
Yes	150 (65.7)	2.49 (0.87–7.42)	0.093	280 (71.4)	0.84 (0.40–1.72)	0.644	112 (77.8)	5.88 (0.93–42.49)	0.064
**Complex in vaccine appointment and uptake**									
No	156 (76.8)	1 (ref.)		303 (84.2)	1 (ref.)		118 (86.8)	1 (ref.)	
Yes	47 (23.2)	0.17 (0.04–0.64)	0.004	57 (15.8)	0.63 (0.28–1.47)	0.277	18 (13.2)	0.12 (0.02–0.65)	0.017
**Trust in Chinese media**									
No	80 (35.1)	1 (ref.)		161 (41.1)	1 (ref.)		53 (36.8)	1 (ref.)	
Yes	148 (64.9)	0.35 (0.11–0.99)	0.055	231 (58.9)	1.14 (0.50–2.58)	0.747	91 (63.2)	5.72 (1.12–34.73)	0.043
**Promptness of Chinese social media**									
No	72 (31.6)	1 (ref.)		139 (35.5)	1 (ref.)		54 (37.5)	1 (ref.)	
Yes	156 (68.4)	0.78 (0.25–2.39)	0.661	253 (64.5)	0.85 (0.37–1.92)	0.699	90 (62.5)	0.66 (0.16–2.49)	0.054
**Unsure of physical suitability for vaccine**									
No	170 (74.6)	1 (ref.)		316 (80.6)	1 (ref.)		127 (88.2)	1 (ref.)	
Yes	58 (25.4)	0.53 (0.19–1.42)	0.203	76 (19.4)	0.26 (0.11–0.58)	0.001	17 (11.8)	0.88 (0.15–5.25)	0.883
**Perceived instant side effect of vaccine**									
No	174 (76.3)	1 (ref.)		315 (80.4)	1 (ref.)		131 (91.0)	1 (ref.)	
Yes	54 (23.7)	1.08 (0.30–4.05)	0.904	77 (19.6)	0.75 (0.29–1.94)	0.547	13 (9.0)	0.68 (0.08–5.90)	0.719
**Perceived long-term side effect of vaccine**									
No	174 (76.3)	1 (ref.)		302 (77.0)	1 (ref.)		125 (86.8)	1 (ref.)	
Yes	54 (23.7)	1.38 (0.41–4.77)	0.609	90 (23.0)	2.56 (1.01–7.04)	0.056	19 (13.2)	0.94 (0.17–5.30)	0.947
**Perceived effectiveness of vaccine**									
No	66 (28.9)	1 (ref.)		121 (30.9)	1 (ref.)		32 (22.2)	1 (ref.)	
Yes	162 (71.1)	1.14 (0.36–3.59)	0.818	271 (69.1)	1.62 (0.69–3.84)	0.270	112 (77.8)	15.97 (2.89–113.02)	0.003
**Prevent COVID-19 infected by vaccine**									
No	82 (36.0)	1 (ref.)		153 (39.0)	1 (ref.)		50 (34.7)	1 (ref.)	
Yes	146 (64.0)	0.59 (0.20–1.65)	0.325	239 (61.0)	1.00 (0.46–2.12)	0.992	94 (65.3)	1.78 (0.35–8.96)	0.478
**Prevent progression of COVID-19 to severe by vaccine**									
No	70 (30.7)	1 (ref.)		110 (28.1)	1 (ref.)		27 (18.8)	1 (ref.)	
Yes	158 (69.3)	3.93 (1.35–12.30)	0.014	282 (71.9)	1.38 (0.61–3.07)	0.434	117 (81.2)	0.21 (0.02–1.5)	0.147

^*^Age between 35–44 and 45–71 were combined as one group because original two groups cannot conduct regression analysis for insufficient sample size of each group.

## Discussion

The vaccination prevalence among migrants from 109 countries (72.9%) was comparable to that among Chinese nationals (77.6%) (36). Moreover, the COVID-19 vaccination rates of some migrants and ethnic minorities in European countries are significantly low (37). Although the vaccination rate among foreign migrants in China was higher than that among migrants in Europe, from the perspective of the total goal of maximizing vaccination coverage, there is still a prospect of promoting vaccination coverage among migrants in China. The price of one dose of the COVID-19 vaccine is ~US$15 for foreign migrants in Beijing (26), Shanghai (27), Guangdong (28), Zhejiang (29), and Wuhan (30), and a previous study suggested over 80% of migrants were willing to pay US$15 for the vaccine (38). Foreign migrants are allowed to be vaccinated in China from April 2021. Foreign migrants who are eligible for taking vaccines may make the vaccination appointment through the following ways: (1) if foreign nationals are employed, they can make the appointment through the employer, and their employer will collect all the information and book for its employees with the local health department; (2) individuals can make the appointment through their residential community (village) offices, and the latter will book with the local health department; and (3) individuals can book the vaccination appointment directly with the local designated hospitals (29). When the proportion of migrants willing to pay for the vaccine is greater than the number actually receiving it, more non-economic incentives should be provided to increase the rate of vaccination among migrants. Although the coverage of COVID-19 vaccination seems sufficient among migrants in China, health inequity was present among migrants of countries with different economic levels; migrants from Asian countries (emerging and developing Asian and Middle Eastern and Central Asian countries) showed a higher COVID-19 vaccination rate than those from G7 countries. Globally, it is common for low-income countries to face COVID-19 vaccine shortages (39). Regarding migrants from low-income countries in this study, although their vaccination rate in China is relatively low compared to that in middle- and high-income countries, their actual vaccination rate (64.7%) is still much higher than the vaccination rate in low-income countries worldwide (19.1%) (31). High-income countries have successfully eliminated numerous vaccine-preventable diseases. Consequently, many people may not recognize the importance of vaccines because they have not seen the devastating effects of some diseases (40). Migrants' concerns about vaccines may go beyond their close social relationships and affect vaccine attitudes and behavior in their countries of origin (41). Cultural differences may also contribute to explaining this phenomenon, which can be explored by Hofstede's cultural dimensions theory in a way. Those cultures inclined to rigid adherence to rules and risk aversion may favor mandates or measures that could be immediately embodied in behavioral changes that are shown to be effective (42).

Long-term orientation countries tend to control gratification as opposed to short-term orientation countries, which prefer “living for today” (43). The citizens living in the former are willing to give up their needs to enjoy the moment and cooperate with the government, which means that the countries that choose restraint tend to demonstrate more effective efforts to curb COVID-19 infections. In contrast, citizens living in the latter countries may need to sacrifice their opportunity costs to achieve the same policy effects as long-term orientation countries, displaying difficulties in controlling the COVID-19 pandemic (44). Therefore, emerging and developing Asian economies have higher vaccination rates than G7 countries. Moreover, populations with high religious uniformity have higher COVID-19 vaccination rates, such as among Native Americans (45). On account of returning to their community events as soon as possible and preserving religious traditions and cultures, community members will be continuously motivated to get vaccinated by the internal forces of religion and society will follow. This kind of impetus in religious communities or ethnic minorities may play a similar role in the Middle East and Central Asia. Migrants with higher educational levels had stronger awareness of COVID-19, better health literacy, higher trust in healthcare professionals, and more interaction with these professionals (5, 46). Therefore, they were less inclined to have vaccine hesitancy and had high vaccination uptake (42).

The 5As model affords comprehensive insights into the determinants of vaccine uptake among migrants. The present study found that active social participation, an access determinant of vaccine uptake, was associated with higher vaccination uptake. Cultural differences may hinder migrants from adopting health behaviors and positive attitudes toward preventive health care, including the importance of immunization (47). Social participation can help bring targeted health education measures to migrant communities, while culturally adapted education could empower migrants to take up vaccines (48). Social participation contributes to health behaviors. Promoting social participation could be an important strategy for community health promotion (49, 50). Furthermore, migrants with better acculturation competence are more willing to get vaccinated (51). The complexity of vaccine appointments and uptake as a convenience factor of affordability was significantly associated with vaccine uptake, which was consistent with previous studies (24, 52). It is beneficial to promote vaccination by simplifying or streamlining the appointment process (8).

Among awareness determinants, the present study found that the awareness of physical suitability for vaccination was associated with vaccine uptake. To some degree, worries over one's physical suitability for vaccination are relevant to their physical constitution or previous medical experiences (53). Previous research has shown that concerns about vaccines being inadequate for one's physical condition may cause vaccine rejection (54). This is not unique. A study on COVID-19 vaccination willingness among Chinese residents reached a similar conclusion: the possibility of being vaccinated among those who thought their physical condition was good enough for vaccination was higher among others (55). Preventing the progression of COVID-19 by vaccination is an important part of the perceived benefits of vaccination and is strongly associated with vaccine uptake (32, 56). People are more likely to get vaccinated when their perceived benefits of vaccines are higher (57). Especially for new vaccines such as the COVID-19 vaccine, people may pay more attention to the expected benefits (58, 59).

In conclusion, promoting migrant vaccination coverage is a global health affair; therefore, the WHO put forward priority actions to achieve high confidence and uptake of COVID-19 vaccines among migrants (9). For example, social coordination mechanisms and policy planning should be maintained and improved. In addition, regular government-led advocacy, communication, and social mobilization activities should be actively conducted (9). By encouraging migrant residents to participate in the construction of their community, their social trust and cohesion will be increased (60).

Importantly, the present study described the status of COVID-19 vaccination and its distribution among foreign migrants in China and provided insight into the 5As model for explaining the determinants of COVID-19 vaccine uptake. There are some potential limitations to this study. First, due to convenience and snowball sampling, selection bias, such as the participation of fewer respondents with low Chinese/English language ability, may have affected the generalizability of the results. Second, the previous studies showed that determinants of receiving primary vaccination series and booster dose might be different (61, 62); however, cluster analysis cannot be performed according to a different number of doses because this information was not included in the present study. Furthermore, the cross-sectional study design could not calculate the causal relationship between vaccine uptake and its determinants. Lastly, the present study collected self-reported data; therefore, social desirability bias and recall bias may underestimate or overestimate the coefficient.

## Data availability statement

The original contributions presented in the study are included in the article/Supplementary material, further inquiries can be directed to the corresponding author.

## Ethics statement

The present study was approved by the Institutional Review Board of East China Normal University Committee on Human Research Protection (HR 161-2021). The patients/participants provided their online written informed consent to participate in this study.

## Author contributions

FW conceived the project and the guarantor of the paper. FW and WL designed the study protocol and carried out data collection. FW and HC conceptualized the current analysis. FW, HC, WL, and ZW discussed and analyzed the data and drafted and revised the manuscript. All authors have read and approved the final manuscript.
