# Supplementary material for: The determinants of COVID-19 vaccine uptake among migrants from 109 countries residing in China: A cross-sectional study
Source: Front Public Health. 2023 Jan 16;10:1023900. doi: 10.3389/fpubh.2022.1023900 (PMC9884687; doi:10.3389/fpubh.2022.1023900)
Supplement: Supplementary file 1 [file Table_1.DOCX]

Table S1. Distribution of age, sex and vaccination rate according to each country

| Country | N | Age (years) | Male (%) | Vaccination rate (%) |
| --- | --- | --- | --- | --- |
| Albania | 1 | 30 | 1 (100.0) | 1 (100.0) |
| [Afghanistan](javascript:;) | 2 | 25.5±0.70 | 2 (100.0) | 1 (50.0) |
| Argentina | 6 | 23.3±4.13 | 2 (33.3) | 3 (50.0) |
| Egypt | 13 | 31.5±4.65 | 12 (92.3) | 9 (69.2) |
| Ethiopia | 4 | 24.25±2.63 | 4 (100.0) | 3 (75.0) |
| Ireland | 1 | 30 | 1 (100.0) | 1 (100.0) |
| [Australia](javascript:;) | 2 | 30.50±6.36 | 0 | 1 (50.0) |
| Pakistan | 101 | 28.62±3.55 | 87 (86.1) | 93 (92.1) |
| Panama | 2 | 19.0±2.83 | 1 (50.0) | 1 (50.0) |
| Brazil | 9 | 28.11±7.82 | 5 (55.6) | 5 (55.6) |
| Belarus | 1 | 23 | 0 | 0 |
| Bulgaria | 1 | 51 | 1 (100.0) | 1 (100.0) |
| [North Macedonia](javascript:;) | 1 | 20 | 0 | 0 |
| Bernin | 2 | 28.0±8.49 | 2 (100.0) | 2 (100.0) |
| Belgium | 1 | 24 | 1 (100.0) | 1 (100.0) |
| Poland | 4 | 44.5±6.76 | 2 (50.0) | 4 (100.0) |
| [Bolivia](javascript:;) | 1 | 21 | 1 (100.0) | 0 |
| [Burundi](javascript:;) | 10 | 30.2±4.30 | 9 (90.0) | 7 (70.0) |
| North [Korea](javascript:;) | 1 | 21 | 1 (100.0) | 1 (100.0) |
| [Equatorial Guinea](javascript:;) | 3 | 23.0±1.0 | 3 (100.0) | 3 (100.0) |
| Germany | 15 | 41.87±14.22 | 9 (60.0) | 12 (80.0) |
| Togo | 5 | 28.60±4.34 | 4 (80.0) | 3 (60.0) |
| Dominican | 1 | 19 | 1 (100.0) | 1 (100.0) |
| [Russia](javascript:;) | 15 | 29.27±5.06 | 10 (100.0) | 7 (46.7) |
| Ecuador | 5 | 28.6±2.41 | 1 (20.0) | 5 (100.0) |
| [Eritrea](javascript:;) | 1 | 42 | 1 (100.0) | 1 (100.0) |
| [France](javascript:;) | 11 | 33.09±7.80 | 8 (72.7) | 7 (63.6) |
| Philippines | 6 | 30.67±9.75 | 0 | 4 (66.7) |
| Finland | 3 | 32.33±2.52 | 3 (100.0) | 0 |
| Gambia | 4 | 21.0±1.15 | 4 (100.0) | 4 (100.0) |
| Democratic Republic of the Congo | 2 | 24.50±6.36 | 17 (68.0) | 2 (100.0) |
| Republic of the Congo | 25 | 24.96±3.30 | 2 (100.0) | 13 (52.0) |
| Colombia | 21 | 29.57±8.42 | 3 (14.3) | 18 (85.7) |
| [Costa Rica](javascript:;) | 2 | 31.0±4.42 | 1 (50.0) | 1 (100.0) |
| Cuba | 1 | 22 | 1 (100.0) | 1 (100.0) |
| [Guyana](javascript:;) | 1 | 27 | 1 (100.0) | 1 (100.0) |
| Kazakhstan | 2 | 25.0±1.41 | 2 (100.0) | 2 (100.0) |
| [Haiti](javascript:;) | 1 | 22 | 1 (100.0) | 1 (100.0) |
| South Korea | 37 | 26.95±7.06 | 10 (27.0) | 11 (29.7) |
| Netherlands | 2 | 45.0±5.66 | 2 (100.0) | 1 (50.0) |

Table S1 (continued). Distribution of age, sex and vaccination rate according to each country

|  | N | Age (years) | Male (%) | Vaccination rate (%) |
| --- | --- | --- | --- | --- |
| [Guinea](javascript:;) | 2 | 27.0±7.07 | 2 (100.0) | 1 (50.0) |
| Canada | 13 | 34.46±13.25 | 9 (69.2) | 9 (69.2) |
| Ghana | 11 | 27.82±13.25 | 9 (81.8) | 9 (81.8) |
| Gabon | 5 | 23.20±2.28 | 2 (40.0) | 4 (80.0) |
| Cambodia | 2 | 27.50±9.19 | 0 | 2 (100.0) |
| Zimbabwe | 13 | 24.15±2.58 | 13 (100.0) | 6 (46.2) |
| Cameroon | 3 | 27.33±1.15 | 2 (66.7) | 1 (33.3) |
| [Comorin](javascript:;) | 1 | 23 | 1 (100.0) | 1 (100.0) |
| [Cote d'Ivoire](javascript:;) | 3 | 24.0±2.0 | 3 (100.0) | 3 (100.0) |
| Kenya | 6 | 23.67±1.63 | 3 (50.0) | 4 (66.7) |
| [Latvia](javascript:;) | 2 | 42.0±8.49 | 0 | 1 (50.0) |
| [Laos](javascript:;) | 6 | 27.0±3.58 | 5 (83.3) | 5 (83.3) |
| [Lebanon](javascript:;) | 2 | 23.0±2.82 | 1 (50.0) | 2 (100.0) |
| [Liberia](javascript:;) | 1 | 23 | 1 (100.0) | 1 (100.0) |
| [Libya](javascript:;) | 1 | 35 | 0 | 1 (100.0) |
| [Rwanda](javascript:;) | 7 | 29.43±2.82 | 7 (100.0) | 3 (42.9) |
| [Madagascar](javascript:;) | 2 | 26.0±2.83 | 0 | 0 |
| [Malawi](javascript:;) | 1 | 25 | 1 (100.0) | 1 (100.0) |
| [Malaysia](javascript:;) | 2 | 27.5±9.19 | 1 (50.0) | 2 (100.0) |
| [Mali](javascript:;) | 8 | 31.88±10.63 | 8 (100.0) | 5 (62.5) |
| [America](javascript:;) | 44 | 35.21±14.03 | 25 (56.8) | 35 (79.5) |
| [Bangladesh](javascript:;) | 39 | 26.59±4.42 | 36 (92.3) | 32 (82.1) |
| [Peru](javascript:;) | 4 | 35.75±15.73 | 1 (25.0) | 3 (75.0) |
| [Burma](javascript:;) | 1 | 25 | 0 | 0 |
| [Morocco](javascript:;) | 3 | 25.0±6.0 | 0 | 2 (66.7) |
| [Mozambique](javascript:;) | 1 | 28 | 1 (100.0) | 1 (100.0) |
| [Mexico](javascript:;) | 9 | 28.2±7.16 | 2 (22.2) | 6 (66.7) |
| [Namibia](javascript:;) | 2 | 36.0±5.66 | 2 (100.0) | 2 (100.0) |
| [South Africa](javascript:;) | 9 | 31.56±6.04 | 3 (33.3) | 7 (77.8) |
| [South Sudan](javascript:;) | 3 | 23.33±0.58 | 3 (100.0) | 2 (66.7) |
| [Nepal](javascript:;) | 4 | 25.25±1.26 | 4 (100.0) | 3 (75.0) |
| Niger | 6 | 29.0±8.12 | 6 (100.0) | 4 (66.7) |
| [Nigeria](javascript:;) | 8 | 25.38±4.37 | 6 (75.0) | 5 (62.5) |
| [Portugal](javascript:;) | 3 | 25.67±2.52 | 2 (66.7) | 2 (66.7) |
| Japan | 10 | 31.7±8.23 | 7 (70.0) | 8 (80.0) |
| [Serbia](javascript:;) | 3 | 30.67±6.51 | 1 (33.3) | 2 (66.7) |
| Sierra Leone | 4 | 31.0±9.42 | 3 (75.0) | 4 (100.0) |
| [Senegal](javascript:;) | 1 | 27 | 0 | 1 (100.0) |
| Saudi Arabia | 1 | 31 | 1 (100.0) |  |
| [Sao Tome and Principe](javascript:;) | 5 | 21.20±1.10 | 5 (100.0) | 5 (100.0) |
| [Slovakia](javascript:;) | 1 | 36 | 0 | 1 (100.0) |
| [Sudan](javascript:;) | 4 | 34.25±7.80 | 4 (100.0) | 2 (50.0) |

Table S1 (continued). Distribution of age, sex and vaccination rate according to each country

|  | N | Age (years) | Male (%) | Vaccination rate (%) |
| --- | --- | --- | --- | --- |
| [Thailand](javascript:;) | 7 | 24.86±5.49 | 6 (85.7) | 3 (42.9) |
| [Tanzania](javascript:;) | 13 | 26.86±5.49 | 9 (69.2) | 8 (61.5) |
| [Tonga](javascript:;) | 1 | 24 | 0 | 1 (100.0) |
| Turks and Caicos Islands | 1 | 38 | 1 (100.0) | 1 (100.0) |
| [Turkey](javascript:;) | 7 | 35.14±11.73 | 5 (71.4) | 5 (71.4) |
| [Turkmenistan](javascript:;) | 10 | 21.10±11.73 | 7 (70.0) | 6 (60.0) |
| [Guatemala](javascript:;) | 2 | 29.50±6.36 | 1 (50.0) | 2 (100.0) |
| [Venezuela](javascript:;) | 7 | 29.0±5.38 | 2 (28.6) | 4 (57.1) |
| [Uganda](javascript:;) | 5 | 26.0±5.34 | 4 (80.0) | 4 (80.0) |
| [Ukraine](javascript:;) | 3 | 26.67±3.79 | 2 (66.7) | 3 (100.0) |
| [Uzbekistan](javascript:;) | 8 | 25.25±4.33 | 7 (87.5) | 5 (62.5) |
| Spain | 5 | 36.40±14.10 | 5 (100.0) | 5 (100.0) |
| [Greece](javascript:;) | 1 | 25 | 0 | 0 |
| [Singapore](javascript:;) | 4 | 25.25±4.72 | 3 (75.0) | 2 (50.0) |
| [New Zealand](javascript:;) | 3 | 35.33±7.23 | 3 (100.0) | 1 (33.3) |
| [Yemen](javascript:;) | 13 | 30.23±5.05 | 11 (84.6) | 12 (92.3) |
| [Iran](javascript:;) | 4 | 26.0±6.48 | 3 (75.0) | 3 (75.0) |
| [Italy](javascript:;) | 6 | 32.83±12.70 | 4 (66.7) | 6 (100.0) |
| [India](javascript:;) | 14 | 34.07±11.89 | 10 (71.4) | 14 (100.0) |
| [Indonesia](javascript:;) | 9 | 25.89±4.34 | 3 (33.3) | 9 (100.0) |
| [England](javascript:;) | 21 | 34.71±10.59 | 15 (71.4) | 12 (57.1) |
| [Jordan](javascript:;) | 1 | 19 | 0 | 1 (100.0) |
| [Vietnam](javascript:;) | 7 | 32.14±3.58 | 2 (28.6) | 7 (100.0) |
| [Zambia](javascript:;) | 16 | 25.69±6.30 | 9 (56.2) | 14 (87.5) |
| [Chad](javascript:;) | 2 | 30.50±3.54 | 2 (100.0) | 1 (50.0) |
| [Chile](javascript:;) | 3 | 43.33±3.51 | 0 | 1 (33.3) |
| Central Africa | 9 | 30.33±6.30 | 9 (100.0) | 5 (55.6) |
